# Cysteic Acid in Dietary Keratin is Metabolized to Glutathione and Liver Taurine in a Rat Model of Human Digestion

**DOI:** 10.3390/nu8020104

**Published:** 2016-02-19

**Authors:** Frances M. Wolber, Michelle McGrath, Felicity Jackson, Kim Wylie, Anne Broomfield

**Affiliations:** 1 School of Food and Nutrition, Massey University, Palmerston North 4442, New Zealand; m.mcgrath@massey.ac.nz (M.M.); yacanto@slingshot.co.nz (K.W.); a.m.broomfield@massey.ac.nz (A.B.); 2 Riddet Innovation, Massey University, Palmerston North 4442, New Zealand; f.s.jackson@massey.ac.nz

**Keywords:** cysteine, keratin, rat model, glutathione

## Abstract

Poultry feathers, consisting largely of keratin, are a low-value product of the poultry industry. The safety and digestibility of a dietary protein produced from keratin (KER) was compared to a cysteine-supplemented casein-based diet in a growing rat model for four weeks. KER proved to be an effective substitute for casein at 50% of the total dietary protein, with no changes in the rats’ food intake, weight gain, organ weight, bone mineral density, white blood cell counts, liver glutathione, or blood glutathione. Inclusion of KER in the diet reduced total protein digestibility from 94% to 86% but significantly increased total dietary cysteine uptake and subsequent liver taurine levels. The KER diet also significantly increased caecum weight and significantly decreased fat digestibility, resulting in a lower proportion of body fat, and induced a significant increase in blood haemoglobin. KER is therefore a safe and suitable protein substitute for casein, and the cysteic acid in keratin is metabolised to maintain normal liver and blood glutathione levels.

## 1. Introduction

Keratin is a major component of poultry feathers and has long been used as a source of dietary protein in animal feeds [1]. However, inclusion of feather meal at a concentration >10% of the diet has been shown to significantly impair growth, weight gain, and food intake [2,3,4]. This is because the keratin protein in feather meal is poorly digestible [4,5,6]. Small amounts of feather meal in animal diets have been shown to have little negative effect on health [3,7,8,9,10].

A benefit of keratin is the high concentration of cysteine (cys) present in feathers. Cys is a semi-essential amino acid in mammals and is an important rate-limiting factor for the synthesis of glutathione, the most important and abundant anti-oxidant in the body [11]. There is evidence that altering dietary concentrations of the sulphated amino acids (SAA) cys and methionine (met) can affect glutathione and its dependent enzymes in specific organs [12,13], with the liver being most strongly affected by dietary protein SAA [14].

While the keratin in rendered feather meal is a poor protein source, hydrolysis or other treatment of poultry feathers may produce a more bioavailable protein. However, increasing the digestibility of a protein through hydrolysis treatment may reduce the concentrations of cys present [15,16]. Thus, there would be value in producing a dietary protein from feather meal that both is highly digestible and retains a high cys content.

A keratin product (KER) was selected for study for which the poultry feathers had been treated using a proprietary method with minimal hydrolysis to create a more bioavailable, high-cys protein source. KER had a reported *in vitro* digestibility of 84%–90% using a standard pepsin digestion method, but its digestibility had not been verified *in vivo*. It has been demonstrated that *in vitro* digestibility may not accurately predict *in vivo* digestibility [17]. In addition, the digestibility of individual amino acids can differ from total protein digestibility and may be influenced by the hydrolysis method [15]. The cys in KER is largely in the form of cysteic acid, which is normally considered to be poorly digestible [18]. As keratin in any form is not a common component of the human diet, and the safety and health effects of crude or purified keratin in humans is unknown, the KER product required assessment in an animal model.

A rat model of *in vivo* digestibility was used to determine whether feeding KER allowed SAA to be absorbed by the gut, retained in the body, translocated to the blood and liver, and incorporated into glutathione. KER as a partial dietary protein source was compared to a control diet containing casein, a dietary protein common in the human diet. A diet comprised solely of KER was not assessed as such a diet is unlikely to be acceptable for human consumers. To ensure that significant changes in protein absorption and tissue glutathione concentrations were measurable, a third group of rats were fed a diet formulated from yellow pea (Pisum sativum) flour (PEA). PEA is severely deficient in the essential amino acid met and thus the diet would provide insufficient SAA for the rats’ dietary needs [19,20,21,22].

KER proved to be a safe, suitable replacement for casein as 50% of the dietary protein. The cys in KER was both digestible and functional. Of interest, the KER-containing diet improved liver taurine and blood haemoglobin levels.

## 2. Materials and Methods

### 2.1. Diets

The proprietary keratin product (KER) was supplied by Keraplast Research Ltd (Christchurch, NZ; patent application number US 13/381,766). KER is produced by oxidising insoluble keratin at low pH with heat, and then raising the pH to form a solution of high molecular weight proteins in a low salt solution. Sodium caseinate (CAS) was purchased from Tatua (Morrinsville, NZ). Yellow pea flour was purchased from Namaste Food and Spices (Auckland, NZ). Dietary components were analyzed by the International Accrediation New Zealand (IANZ)-accredited Massey University Nutrition Laboratory. Components of protein sources (Table 1) were determined by the following methods: nitrogen, Leco total combustion method (AOAC (Association of Official Agricultural Chemists International) 968.06), with CAS protein calculated as 6.38 X nitrogen, and KER and PEA protein calculated as 6.25X nitrogen; fat, Soxtec extraction (AOAC 991.36); moisture, convection oven drying at 105 °C (AOAC 930.15, 925.10); ash, 550 °C furnace (AOAC 942.05); gross energy, bomb calorimetry; amino acids, hydrochloric acid analysis followed by HPLC separation (AOAC 994.12); tryptophan, alkaline hydryolysis followed by HPLC separation; cysteic acid, hydrochloric acid hydrolysis and HPLC separation; total cys, performic acid oxidation, with total native cysteine calculated by subtracting cysteic acid from total cys, normalised for their respective molar weights. Carbohydrate was taken to be the total minus protein, fat, ash, and moisture.

Dietary ingredients l-cysteine, methionine, glutamic acid, glycine, lysine, calcium carbonate, potassium phosphate, potassium sulphate, potassium citrate, and magnesium oxide were purchased from Merck (Darmstadt, Germany). Tryptophan, ferric citrate, manganous sulphate, zinc oxide, cupric carbonate, chromic potassium sulphate, sodium selenite, cobaltous chloride, potassium iodate, and ammonium molybdate were purchased from Sigma-Aldrich (Auckland, NZ). Cellulose was purchased from Hawkins Watts (Auckland, NZ). Cornstarch, soy oil, and sucrose were purchased from Davis Trading (Palmerston North, NZ). Vitamin mix (Unitech; Auckland, NZ) and sodium-free mineral mix were prepared to American Institute of Nutrition (AIN)-96G specifications.

Diets were formulated (Table 2) to contain energy at 17 kJ/g and to comprise 7% fat, 10% fibre, 0.34% sodium, and 17% protein from the desired source (CAS, CAS + KER, or PEA). All diets were designed to meet AIN-93G amino acid and micronutrient requirements specifications, with the exception of SAA: the CAS diet was formulated to contain cys at 3.3 g/kg and met at 6.5 g/kg from the combination of CAS and free amino acids; the KER + CAS diet to contain cys at 5.3 g/kg and met at 6.5 g/kg from the combination of CAS + KER and free amino acids; the PEA diet to contain cys at 3.3 g/kg and met at 1.7 g/kg from the combination of PEA and free amino acids. Diets were labelled, colour-coded, and stored at −20 °C.

### 2.2. Animal Study

All procedures were carried out with the approval of the Massey University Animal Ethics Committee (approval #11/16) and followed national and international guidelines for the care and use of animals. Conventional male Sprague–Dawley rats aged 5 weeks were obtained from the Massey University Small Animal Production Unit and individually housed in plastic cages with wire lids, bedded with sterile wood shavings in a room maintained at 22 ± 0.5 °C with a 12 h light/dark cycle. All rats had access to food and water *ad libitum*. Rats were fed standard rat chow for the first 3 days of acclimatisation to their new housing. Rats were then randomised into test groups by body weight (CAS, 195 ± 10 g; KER + CAS, 196 ± 9 g; PEA, 200 ± 3 g), and fed the appropriate test diet. Food intake was measured daily, and body weight twice weekly.

From days 5 to d 11 of the study, rats were placed in metabolic cages. After 2 days acclimatisation, urine and faecal outputs were collected daily for 4 days. Urine samples were acidified with the addition of 0.1 mL 1M HCl to acidify the urine and 0.5 mL of water to rinse the collection tube. Urine and faecal samples for each rat were pooled and stored at −20 °C.

On days 28–30 of the study, rats were deeply anesthetised by i.p. injection of acepromazine, ketamine, and xylazine (AKX) and scanned for body composition using a Dual-Energy X-ray Absorptiometer (DEXA; Hologic model Discovery A). Rats were then killed with additional AKX, exsanguinated, and pneumothorax induced prior to dissection. Blood samples were stored at −80 °C, liver samples snap-frozen in liquid nitrogen and stored at −80 °C, and carcasses stored at −20 °C. Carcasses were later thawed and scanned using the same DEXA system for bone mineral density.

### 2.3. Tissue and Sample Analyses

Blood erythrocyte and liver tissue samples were prepared and assayed for total, reduced (GSH), and oxidized (GSSG) glutathione as per kit instructions (Glutathione Assay Kit; Cayman Chemical Company, Ann Arbor, MI, USA) in duplicate wells per sample. Optical density readings of each well were normalised to a seven-point standard curve. GSH was calculated as (total glutathione) − (2 × GSSG).

Complete blood counts (CBC) and haemoglobin analyses on whole blood were carried out by NZ Veterinary Pathology (Palmerston North, NZ). Tissues were analyzed by the IANZ-accredited Massey University Nutrition Laboratory using the following assays: cys (cysteine + cystine), met and taurine in heparinised whole blood and snap-frozen livers liver by performic acid oxidation and hydrochloric acid analysis followed by HPLC separation (AOAC 994.12); nitrogen in urine and in dried, ground, sifted faeces by Leco total combustion method (AOAC 968.06); creatine in urine by the Jaffe method; fat in faeces by Soxtec extraction (AOAC 991.36); dry matter in faeces by convention oven drying at 105 °C (AOAC 930.15, 925.10); ash in faeces by furnace treatment at 550 °C (AOAC 942.05); amino acids in faeces by hydrochloric acid hydrolysis followed by HPLC separation (AOAC 994.12).

Digestibility was calculated as D = (IP − FP) × 100/IP, where D = apparent digestibility, IP = ingested protein, and FP = faecal protein. Faecal protein was calculated as N × 6.25.

### 2.4. Statistical Analyses

Means and standard deviations were calculated for each group and statistical analyses carried out using one-way ANOVA followed by Bonferonni *post hoc* testing (Primer of Biostatistics version 3.02; McGraw-Hill, Inc., Columbus, OH, USA), with *p* ≤ 0.05 being considered significant.

## 3. Results

Rats were fed one of three test diets (CAS, KER + CAS, and PEA; *n* = 10 per group) for four weeks. Rats did not differ by group in food intake by volume (Table 3), although PEA rats had a lower energy intake and a lower body weight gain.

At the end of the study, both PEA and KER + CAS rats had lower proportional body fat than the CAS control rats, although this did not reach statistical significance. The KER + CAS diet did not adversely affect bone mineral density (BMD) or bone mineral content (BMC); PEA rats had slightly lower BMD and BMC.

The liver, kidneys, spleen, and caecum were dissected out of each rat and weighed, and organ weights normalised to body weight (BW). There were no differences between groups in the weights of liver, kidneys, or spleen (data not shown). However, both KER + CAS and PEA groups had significantly heavier caecum weights (12.4 ± 3.3 and 22.0 ± 5.4 g/kg BW, respectively; *p* ≤ 0.05 by ANOVA) compared to CAS (7.5 ± 1.3 g/kg BW). Caecum size was reflected in faecal moisture. Both KER + CAS and PEA rats produced faeces with significantly more moisture (47.9% ± 3.0% and 52.8% ± 2.9%, respectively; *p* ≤ 0.05 by ANOVA) compared to CAS (33.3% ± 2.1%) rats.

Complete blood counts (CBC) were performed on peripheral blood samples. The PEA diet did not affect red or white cell counts or haemoglobin concentrations (Table 4). In contrast, the KER + CAS diet increased RBC counts and significantly increased total haemoglobin concentrations.

Protein digestibility and SAA sufficiency in the rats were also assessed. CAS diet contained sufficient amounts of both cys and met and had a suitable cys:met ratio; 85% of the cys was in the form of a free amino acid, and none in the form of cysteic acid. The KER + CAS diet contained sufficient met and excess cys, and had a suitable cys:met ratio. The cys was present as 92% cysteic acid, with the remainder coming from dietary protein. The PEA diet was deficient in met and contained a cys:met ratio that did not meet the rats’ dietary needs, but contained no cysteic acid. To determine whether the increased concentration of SAA in the form of cysteic acid in the KER diet resulted in a higher absorption and retention of SAA, tissues, faeces and urine were collected from rats held in metabolic cages for a 4 days period and analyzed.

Mean daily urine output, total 4 days urine output, urine creatinine output, and urine nitrogen output did not vary significantly between test groups (data not shown). Rats fed the PEA diet ate less food and excreted less faecal material (Table 5). Fat ingestion did not differ between groups, but both PEA and KER + CAS rats excreted more fat compared to control, reducing total fat digestibility. Protein ingestion likewise did not differ between groups, while protein excretion did differ significantly, resulting in significantly lower apparent digestibilities for the protein in those diets. The digestibility of the KER + CAS protein diet was 86%; if the digestibility of casein in the KER diet remained at 94% as observed in the CAS diet, the digestibility of KER itself would be calculated as 78%.

Total protein intake and digestibility for each dietary protein source was not consistently reflected in the intake and digestibility of individual amino acids. As expected based on the diet compositions, rats on the PEA diet ingested significantly less SAA, while rats on the KER + CAS diet ingested significantly more cys. Some of the excess cys in the KER + CAS diet was excreted, resulting in a significantly lower apparent digestibility for cys (measuring cysteine + cystine combined) in this diet, but KER + CAS rats absorbed significantly more total cys than CAS control rats. Met was excreted rather than absorbed in PEA rats, despite this diet being deficient in met.

To determine whether cysteic acid absorbed from the KER + CAS diet maintained sufficient glutathione and SAA levels in the rats, total glutathione, reduced glutathione (GSH), and oxidised glutathione (GSSG) were measured in the liver and in peripheral blood erythrocytes. There were no significant differences observed between KER + CAS and the CAS control groups in glutathione, GSH, GSSG, or GSH:GSSG ratios in the liver or blood (Table 6). PEA rats had significantly less liver glutathione and liver GSH, although blood concentrations did not differ. PEA rats likewise had significantly lower concentrations of liver cys and taurine. In contrast, KER + CAS rats had significantly higher liver taurine.

## 4. Discussion

The current study examined the effect of replacing 50% of the casein in a nutritionally complete rat diet with the keratin product KER, which contains a high proportion of cysteic acid. KER proved to be a suitable substitute for CAS at up to 50% of the total protein in the diet. Rats fed the KER + CAS diet ate the same amount as those fed the CAS diet, indicating that there were no problems with palatability of the KER diet. Weight gain was similarly unaffected; thus, the KER + CAS diet was adequate to meet the needs of the growing rat and did not contain significant concentrations of anti-nutrients. Rats fed the KER + CAS diet did not differ significantly from rats fed the CAS control diet in organ weight, bone mineral density or bone mineral content, white blood cell counts, liver or blood glutathione, or liver cys. Importantly, KER + CAS resulted in significantly lower fat absorption and significantly increased caecum weight, total cys absorption, blood haemoglobin concentration, and liver taurine, In contrast, rats fed the pea flour-based PEA diet deficient in sulphated amino acids demonstrated significant decreases in weight gain and in liver glutathione, cys, and taurine concentrations.

For ethical reasons, the current study did not include a group of rats fed zero protein to provide a measure of metabolic faecal protein, and thus measured apparent digestibility rather than true digestibility [17]; however, this is sufficient for comparative purposes between protein sources. The digestibility of the protein in the CAS diet in the current study was 94%; other groups have similarly reported similar digestibility values for CAS and other milk proteins [17,23]. The digestibility of the KER + CAS protein diet was 86%, with the digestibility of KER alone calculated to be 78%. This is slightly lower than the reported *in vitro* digestibility of 85.8%–90.4% [24]. It is possible that KER digestibility *in vivo* is >78%, and that the inclusion of KER in the diet reduced the bioavailability of the CAS protein. The inclusion of feather meal in the diet of rainbow trout has been reported to reduce the efficiency of utilisation of digestible crude protein [25].

Individual amino acid digestibilities were not identical to total protein digestibilities within or between diets; variability between amino acid uptakes, and even between isoforms of a given amino acid, have been shown elsewhere [15,26,27]. Much of the excess cys in the KER + CAS diet remained unabsorbed. Free cys concentrations are regulated by the liver [28]; physiological processes limit the uptake of cys as excess cys can be neurotoxic [29,30]. The KER + CAS rats absorbed sufficient met; as the PEA rats failed to absorb met even though their diet was met-deficient, this demonstrates that in the PEA diet, but not the KER + CAS diet, an altered cys:met ratio negatively impacted SAA absorption. It is unclear why the PEA rats excreted rather than absorbed met; however, as the PEA rats excreted a significantly higher proportion of their dietary protein overall, the effect likely extended to other amino acids and thus may reflect poor protein absorption as a result of methionine deficiency. However, this has been reported to not be the case in chicks [31]. It would be of interest in a subsequent study to measure the plasma and faecal concentrations of all amino acids.

Cys is a necessary component of glutathione, a key antioxidant in the body [11]. Cystine is present at a much higher concentration than cysteine in the plasma, whereas cystine is converted intracellularly to cysteine [32,33]. The cys content of KER was mainly in the form of cysteic acid; formation of cysteic acid in keratin has been observed after oxidative treatment [34]. l-cysteine is not an essential amino acid in the rat, which can synthesise it from l-methionine via a trans-sulphuration pathway, but when it is present in the diet, it can spare the l-methionine requirement [35]. Cysteic acid has been reported to have no dietary cys-sparing activity [26] as it is a less digestible form of cys [18], and cysteic acid has been shown to be less able to support weight gain in the growing rat compared to l-cysteine [35]. However, the KER + CAS rats had liver glutathione, cys, and met levels equally as high as the CAS control rats. In contrast, rats fed the SAA-deficient PEA diet had significantly lower liver glutathione and cys. The glutathione concentrations observed in the current study fit with reported concentrations in the normal rat [12,36].

Taurine, a sulfonic acid, is rapidly synthesised in the liver from cys [37]. It is a key component of bile salts and is involved in a variety of physiological functions [38]. Blood taurine concentrations of rats in the current study were negligible, as has been reported elsewhere [39], but did not differ between groups. In both KER + CAS and CAS control groups, liver taurine concentrations were within the normal range for male rats [40]; however, liver taurine was significantly higher in KER + CAS rats compared to CAS control, and significantly lower in PEA rats compared to CAS control. As cysteic acid has been shown to be metabolised to taurine in the rat [34], it is likely that the high concentration of cysteic acid in the KER protein effected the observed increase in liver taurine concentrations. Taurine and cys-containing compounds have hepatoprotective effects [41,42,43].These findings confirmed that the PEA diet was insufficient in SAA, while the KER + CAS diet was superior to the CAS control diet.

The KER + CAS diet produced an additional health benefit by significantly increasing blood haemoglobin levels. RBC counts were also elevated, though the CBC values for each parameter remained within published norms [44]. In a human study, supplementation with *N*-acetylcysteine induced erythropoietin secretion and significantly increased blood haemoglobin [45], confirming the likelihood that the high cys levels in KER were responsible for the observed changes in haemoglobin.

Both PEA and KER + CAS rats had significantly higher caecum weights. In the PEA group, this was likely due to the high dietary fibre in the pea flour; dietary fibres have been shown to increase rat caecum weight [46]. Some proteins have been shown to have similar functions to dietary fibres [47], and oligosaccharides that affect gut microflora and faecal short-chain fatty acids can also increase caecum weight [48,49]. Thus, KER may contain peptides with fibre-like activity and/or prebiotic oligosaccharides. As diet can have a significant impact on the composition of gut microbiota, it would be of interest to assess the effects of KER + CAS *versus* CAS alone on gut microbiota numbers and diversity.

## 5. Conclusions

The current study demonstrated that KER is suitable as a partial protein replacement for CAS uptake and should be safe for human consumption. KER contains high cys in the form of digestible and functional cysteic acid, and thus can be paired with a cys-deficient protein, obviating the need to include free l-cysteine to balance diets. KER when combined with CAS can induce significant increases in dietary cys absorption, liver taurine, and blood haemoglobin. It will be of interest to determine whether the cysteic acid in KER can correct a physiological cys-deficiency and ablate the oxidative stress that occurs with protein malnutrition, and to explore whether KER increases blood haemoglobin by stimulating erythropoeisis in the bone marrow and, increasing reticulocyte counts in the peripheral blood. It will likewise be of interest to conduct dose response studies to determine the minimum level of KER required in the diet to provide the observed health benefits, and to include assessments of liver function such as serum cholesterol, protein, albumin, aspartate aminotransferase, alanine aminotransferase, and triglycerides to ensure there are no hepatotoxic effects.

## Figures and Tables

**Table 1:** Analysis of sources of dietary protein: sodium caseinate (CAS), keratin (KER), and yellow pea flour (PEA). Data are shown in g/kg, except for energy.

	CAS	KER	PEA
Energy (kJ/g)	22.10	21.40	16.70
Nitrogen	14.43	14.57	3.69
Protein	92.06	91.03	23.05
Fat	0.10	0.55	1.80
Moisture	5.70	1.90	11.10
Ash	3.40	6.35	2.60
Carbohydrate	0.60	0.20	61.50
Sodium	1.23	2.40	0.00
Alanine	2.69	3.64	0.92
Arginine	3.48	5.51	2.00
Aspartic acid	6.41	6.46	2.72
Cysteic acid	(0.00)	(7.35)	(0.00)
Cysteine	(0.25)	(0.19)	(0.31)
Total cys	0.25	5.46	0.31
Glutamic acid	19.03	9.97	3.83
Glycine	1.77	6.38	1.04
Histidine	2.88	0.47	0.66
Isoleucine	4.95	4.62	1.07
Leucine	8.36	6.83	1.70
Lysine	6.91	1.07	1.65
Methionine	2.34	0.41	0.23
Phenylalanine	4.76	3.83	1.18
Proline	9.38	8.69	1.01
Serine	4.44	9.41	1.02
Threonine	3.64	4.21	0.85
Tryptophan	1.19	0.00	0.20
Tyrosine	5.10	1.35	0.82
Valine	6.44	8.23	1.23

**Table 2:** Formulation of test diets (ingredients added at g/kg final diet) containing protein sourced from casein (CAS), 50:50 w/w keratin and casein (KER + CAS), or yellow pea flour (PEA).

	CAS	KER + CAS	PEA
Sodium caseinate	185.0	92.0	0.0
Keratin	0.0	93.0	0.0
Yellow pea flour	0.0	0.0	736.0
Vitamin mix	10.0	10.0	10.0
Na-free mineral mix	50.0	50.0	50.0
NaCl	2.8	0.0	8.7
Soy oil	70.0	69.0	57.0
Sucrose	50.0	50.0	31.0
CaCO_3_	12.5	12.5	12.5
Cysteine	2.8	0.0	1.0
Methionine	2.2	3.9	0.0
glutamic acid	4.7	13.0	11.8
Glycine	2.7	0.0	0.0
Tryptophan	0.0	0.9	0.0
Lysine	0.0	1.8	0.0
Cellulose	96.5	96.5	0.0
Cornstarch	510.8	507.4	82.0

**Table 3:** Food intake, body weight gain, and dual-energy X-ray absorptiometer (DEXA) body scan data from rats after 4 weeks of being fed on test diets containing 18% w/w protein sourced from casein (CAS), 50:50 w/w keratin and casein (KER + CAS), or yellow pea flour (PEA) ^1^.

	CAS	KER + CAS	PEA
28 days food intake (g)	661 (15)	672 (11)	623 (12)
28 days energy intake (MJ)	11.8 ^a^ (0.3)	12.0 ^a^ (0.2)	10.5 ^b^ (0.2)
28 days protein intake (g)	120 (3)	128 (2)	118 (2)
28 days BW gain (g)	196 ^a^ (6)	207 ^a^ (6)	169 ^b^ (5)
DEXA: Total mass (g)	412 (9)	423 (8)	394 (9)
DEXA: Fat mass (g)	67 (3)	62 (5)	56 (3)
Fat (% of total mass)	16.3 (0.7)	14.6 (1.0)	14.3 (0.6)
Whole-body BMD (mg/cm)	142 (2)	145 (1)	139 (1)
Whole-body BMC (g)	9.68 (0.16)	9.90 (0.15)	9.23 (0.15)

^1^ Data are presented as mean (±SE) of *n* = 10. Means in a row with superscripts without a common letter differ, *p* < 0.05 by ANOVA.

**Table 4:** Hematological parameters ^1^ of blood from rats after 4 weeks of being fed on test diets containing 18% w/w protein sourced from casein (CAS), 50:50 w/w keratin and casein (KER + CAS), or yellow pea flour (PEA) ^2^.

	CAS	KER + CAS	PEA
WBC (10^9^/L)	7.13 (0.68)	8.60 (0.89)	9.09 (0.64)
RBC (10^12^/L)	7.12 (0.09)	7.42 (0.10)	7.24 (0.11)
Hemoglobin (g/L)	139 ^a^ (2)	146 ^b^ (1)	140 ^a^ (1)
Hematocrit (mL/L)	405 ^a^ (5)	424 ^b^ (5)	412 ^a^ (4)
MCV (fL)	56.8 (0.8)	57.3 (0.8)	57.3 (0.7)
MCH (pg)	19.7 (0.3)	19.8 (0.3)	19.4 (0.3)

^1^ WBC, white blood cell count; RBC, red blood cell count; MCV, mean corpuscular volume; MHC, mean hemoglobin per RBC; ^2^ Data are presented as mean (±SE) of *n* = 10. Means in a row with superscripts without a common letter differ, *p* < 0.05 by ANOVA.

**Table 5:** Digestibilities of protein and amino acids in rats fed for 4 days in metabolic cages with test diets containing 18% w/w protein sourced from casein (CAS), 50:50 w/w keratin and casein (KER + CAS), or yellow pea flour (PEA) ^1^.

	CAS	KER + CAS	PEA
Ingested			
diet (g)	106 ^a^ (2)	108 ^a^ (2)	97 ^b^ (2)
fat (g)	7.76 (0.15)	7.89 (0.13)	7.50 (0.15)
protein (g)	19.4 (0.4)	20.7 (0.3)	18.4 (0.4)
cys (mg)	350.9 ^a^ (6.6)	573.1 ^b^ (9.6)	321.3 ^c^ (6.6)
met (mg)	691.1 ^a^ (13.0)	702.8 ^a^ (11.8)	164.8 ^b^ (3.4)
Excreted			
faeces, dried (g)	17.4 ^a^ (0.3)	17.3 ^a^ (0.5)	11.8 ^b^ (0.3)
fat (g)	0.18 ^a^ (0.01)	0.29 ^b^ (0.02)	0.41 ^c^ (0.01)
protein (g)	1.17 ^a^ (0.05)	2.87 ^b^ (0.08)	2.98 ^b^ (0.11)
cys (mg)	49.0 ^a^ (4.1)	236.7 ^b^ (5.7)	53.2 ^a^ (2.2)
met (mg)	17.6 ^a^ (1.1)	29.2 ^b^ (0.9)	63.7 ^c^ (3.0)
taurine (mg)	5.8 (0.7)	7.6 (0.5)	6.3 (0.6)
Absorbed			
fat (g)	7.58 (0.14)	7.60 (0.14)	7.08 (0.15)
protein (g)	18.19 ^a^ (0.34)	17.78 ^a^ (0.36)	15.42 ^b^ (0.28)
cys (mg)	301.9 ^a^ (9.0)	336.4 ^b^ (10.7)	268.1 ^c^ (7.7)
met (mg)	673.6 ^a^ (12.4)	673.7 ^a^ (11.8)	10.11 ^b^ (1.6)
Excreted (% of ingested)			
protein	6.0 ^a^ (0.2)	13.9 ^b^ (0.4)	16.2 ^c^ (0.3)
Fat	2.37 ^a^ (0.12)	3.70 ^b^ (0.24)	5.50 ^c^ (0.13)
cys	14.1 ^a^ (1.3)	41.4 ^b^ (1.1)	16.6 ^a^ (0.8)
met	2.5 ^a^ (0.1)	4.2 ^a^ (0.1)	38.5 ^b^ (1.1)
Digestibility (%)			
protein	94.0 ^a^ (0.2)	86.1 ^b^ (0.4)	83.8 ^c^ (0.3)
fat	97.6 ^a^ (0.1)	96.3 ^b^ (0.2)	94.5 ^c^ (0.1)
cys	85.9 ^a^ (1.3)	58.6 (1.1)	83.4 ^a^ (0.8)
met	97.5 ^a^ (0.1)	95.8 ^a^ (0.1)	61.5 ^b^ (1.1)

^1^ Data are presented as mean (+SE) of *n* = 10. Means in a row with superscripts without a common letter differ, *p* < 0.05 by ANOVA.

**Table 6:** Glutathione, GSSG (oxidised glutathione), and GSH (reduced glutathione) in μmol/g tissue, in liver and peripheral blood RBC of rats fed for 4 weeks with test diets containing 18% w/w protein sourced from casein (CAS), 50:50 w/w keratin and casein (KER + CAS), or yellow pea flour (PEA) ^1^.

	CAS	KER + CAS	PEA
Liver			
glutathione	15.56 ^a^ (0.81)	16.67 ^a^ (1.00)	12.14 ^b^ (0.83)
GSSG	2.14 (0.24)	2.26 (0.28)	2.09 (0.24)
GSH	11.28 ^a^ (0.89)	12.16 ^a^ (0.96)	7.95 ^b^ (0.86)
cys	3.23 ^a^ (0.04)	3.16 ^a^ (0.03)	3.05 ^b^ (0.05)
met	3.64 (0.08)	3.63 (0.04)	3.72 (0.06)
taurine	1.14 ^a^ (0.06)	1.44 ^b^ (0.03)	0.18 ^c^ (0.05)
RBC			
glutathione	1.06 (0.11)	1.28 (0.12)	1.26 (0.09)
GSSG	0.23 (0.04)	0.30 (0.07)	0.27 (0.04)
GSH	0.60 (0.09)	0.80 (0.10)	0.71 (0.10)
cys	2.62 (0.04)	2.65 (0.05)	2.65 (0.06)
met	1.89 (0.04)	1.91 (0.06)	1.86 (0.06)
taurine	0.05 (0.00)	0.05 (0.00)	0.05 (0.01)

^1^ Data are presented as mean (+SE) of *n* = 10. Means in a row with superscripts without a common letter differ, *p* < 0.05 by ANOVA.
